# Wealth index association with self-reported oral health between white and non-white older Brazilians

**DOI:** 10.1590/0102-311XEN188122

**Published:** 2023-06-26

**Authors:** Orlando Luiz do Amaral, Maria Laura Braccini Fagundes, Gabriele Rissotto Menegazzo, Jessye Melgarejo do Amaral Giordani

**Affiliations:** 1 Programa de Pós-graduação em Ciências Odontológicas, Universidade Federal de Santa Maria, Santa Maria, Brasil.

**Keywords:** Oral Health, Race Relations, Self Concept, Socioeconomic Factors, Saúde Bucal, Relações Raciais, Autoimagem, Fatores Socioeconômicos, Salud Bucal, Relaciones Raciales, Autoimagen, Factores Socioeconómicos

## Abstract

This cross-sectional study aimed to identify the association between self-reported oral health status and a wealth index among white and non-white older adults in Brazil. Data from individual assessments of 9,365 Brazilians aged 50 years or older were analyzed. Poisson regression models were performed to estimate the prevalence ratio between wealth index and self-reported oral health among whites and non-whites adjusted for intermediate and proximal determinants. The total prevalence of poor self-reported oral health on white and non-white individuals was 41.6% (95%CI: 40.0-43.4) and 48% (95%CI: 47.1-49.8) respectively. The adjusted analysis showed that, for whites, the wealth index is associated with self-reported oral health since individuals in the 3rd, 4th, and 5th quintiles have 25% (PR = 0.75; 95%CI: 0.65-0.88), 20% (PR = 0.80; 95%CI: 0.67-0.95), and 39% (PR = 0.61; 95%CI: 0.50-0.75) lower prevalence of poor self-reported oral health than those in the poorest quintile. For non-white individuals, the wealth index is associated with self-reported oral health only for those in the 5th quintile, with 25% (PR = 0.85; 95%CI: 0.72-0.99) lower prevalence of poor self-reported oral health than those in the poorest quintile. The wealth index showed different effects on self-reported oral health among whites and non-whites. Socioeconomic status indicators may reflect racial inequalities due to the historical legacy of institutional discrimination. This study highlights the importance of developing policies to combat racial inequities and how these can contribute to better oral health conditions for the older Brazilian population.

## Resumo

Este estudo transversal teve como objetivo identificar a associação entre o estado de saúde bucal autorreferida e o índice de riqueza entre idosos brancos e não brancos no Brasil. Foram analisados dados de avaliações individuais de 9.365 brasileiros com 50 anos ou mais. Foram utilizados modelos de regressão de Poisson para estimar a razão de prevalência entre o índice de riqueza e a saúde bucal autorreferida entre brancos e não brancos, ajustada para determinantes intermediários e proximais. A prevalência total de autopercepção de saúde bucal ruim em indivíduos brancos e não brancos foi de 41,6% (IC95%: 40,0-43,4) e 48% (IC95%: 47,1-49,8), respectivamente. A análise ajustada mostrou que, para indivíduos brancos, o índice de riqueza está associado à saúde bucal autorreferida para indivíduos do 3º, 4º e 5º quintis com 25% (RP = 0,75; IC95%: 0,65-0,88), 20% (PR = 0,80; IC95%: 0,67-0,95) e 39% (PR = 0,61; IC95%: 0,50-0,75) menor prevalência de saúde bucal autorreferida ruim do que aqueles no quintil mais pobre. Para indivíduos não brancos, o índice de riqueza está associado à saúde bucal autorreferida apenas para aqueles no 5º quintil, com 25% (RP = 0,85; IC95%: 0,72-0,99) menor prevalência de saúde bucal autorreferida ruim do que aqueles no quintil mais pobre. O índice de riqueza mostrou diferentes efeitos sobre a saúde bucal autorreferida entre indivíduos brancos e não brancos. Os indicadores de *status* socioeconômico podem refletir desigualdades raciais devido ao legado histórico da discriminação institucional. Este estudo destaca a importância do desenvolvimento de políticas de combate às iniquidades raciais e como elas podem contribuir para melhores condições de saúde bucal na população brasileira idosa.

## Resumen

Este estudio transversal tuvo como objetivo identificar la asociación entre el estado de salud bucal autorreportada y el índice de riqueza entre ancianos blancos y no blancos en Brasil. Se analizaron datos de evaluaciones individuales de 9.365 brasileños de 50 años o más. Se utilizaron modelos de regresión de Poisson para estimar la relación de prevalencia entre el índice de riqueza y la salud bucal autorreportada entre blancos y no blancos, ajustada para determinantes intermedios y proximales. La prevalencia total de autopercepción de mala salud bucal en individuos blancos y no blancos fue de 41,6% (IC95%: 40,0-43,4) y 48% (IC95%: 47,1-49,8), respectivamente. El análisis ajustado mostró que, para los individuos blancos, el índice de riqueza está asociado con salud bucal autorreportada para individuos en los quintiles 3, 4 y 5 con 25% (RP = 0,75; IC95%: 0,65-0,88), 20% (RP = 0,80; IC95%: 0,67-0,95) y 39% (RP = 0,61; IC95%: 0,50-0,75) menor prevalencia de salud bucal autorreportada mala que aquellos en el quintil más pobre. Para las personas que no son blancas, el índice de riqueza se asocia con salud bucal autorreportada solo para aquellos en el quintil 5, con un 25% (PR = 0,85; IC95%: 0,72-0,99) de menor prevalencia de salud bucal autorreportada mala que aquellos en el quintil más pobre. El índice de riqueza mostró diferentes efectos sobre la salud bucal autorreportada entre individuos blancos y no blancos. Los indicadores de estatus socioeconómico pueden reflejar desigualdades raciales debido al legado histórico de la discriminación institucional. Este estudio destaca la importancia del desarrollo de políticas de combate a las inequidades raciales y cómo ellas pueden contribuir para mejores condiciones de salud bucal en la población brasileña anciana.

## Introduction

Race relations are a complex and lasting social process shaped by an interaction of slavery, class, and gender oppression ^1^. The literature suggests that the racial differences in oral health are large and persistent over time, due to the social structure referring to the patterns of social life that shape the beliefs, attitudes, behaviors, actions, and material and psychological resources of individuals ^2^ ^,^ ^3^. The racial disparities in health have important implications for the development of effective approaches to improve health and reduce inequalities ^4^. In Brazil, more than 5,000,000 slaves were brought to Brazil for almost four centuries ^5^. Brazil was also the last country to abolish slavery in the Western hemisphere, in 1888, making miscegenation a prominent Brazilian demographic characteristic ^6^. Documented evidence shows the worse condition of non-whites compared with those of whites, regarding income, education, labor market, law enforcement, and health conditions ^7^.

Racial inequities can be expressed by different risks of falling ill and dying, originated from heterogeneous conditions of existence and access to health’ goods and services ^8^. Often, socioeconomic status indicators are used to capture social class and refer to social and economic factors that influence the individual’s positions in society and racial inequities ^9^ ^,^ ^10^. Disparities in health status by socioeconomic status are rarely presented by race and socioeconomic status simultaneously ^11^. However, given the patterns of social inequality and the need to raise awareness among the population and policy makers about it, collecting, analyzing, and presenting health data by race is important ^11^, collaborating with the political debate on racial inequalities and the formulation of public oral health policies ^11^.

Race and socioeconomic status combine in complex ways to affect the oral health of the population. However, there is a debate about the desirability of race-specific versus universal initiatives to improve health outcomes for vulnerable social groups ^11^. The residual effects of race, at all levels of the socioeconomic status, may lead to different oral health outcomes, and result in worse coping responses to the health conditions of non-white individuals ^11^ ^,^ ^12^. Research has called attention to large racial/ethnic inequalities in wealth and highlights that these gaps reflect, at least partly, the historical legacy of institutional discrimination ^8^ ^,^ ^13^. Compared with whites, racial minorities have lower income and higher unemployment rates at all levels of education, less wealth at all levels of income, greater exposure to occupational risks and less purchasing power ^8^ ^,^ ^12^.

A previous study suggested that racial disparities can persist in different outcomes, such as tooth loss, perceived oral health, and periodontitis ^14^. In addition, evidence shows that socioeconomic status indicators may reflect racial inequalities, at least partly, due to the historical legacy of institutional discrimination and that these marked disparities can exist at all levels. In Brazilian older adults, the association between skin color and health conditions is controversial and conflicting ^15^. Such conflict has limited the current understanding of the impacts of racial inequalities in this phase of life. Although previous studies have explored the influence of gender, income, schooling, age group, and social context on the health of older adults ^16^ ^,^ ^17^, the association between oral health and wealth indicators in the perspective of skin color/race has not been explored in this age group. Considering these premises, this study aimed to identify the association between self-reported oral health and the wealth index among white and non-white older adults in Brazil. It was hypothesized that, due to the residual effects of race, even non-whites belonging to the wealthiest quintiles would have worse self-reported oral health than whites in the same quintile.

## Methods

The *Strengthening the Reporting of Observational Studies in Epidemiology* (STROBE) guideline was followed to write the manuscript.

### Study design

This cross-sectional study analyzed the baseline data from the *Brazilian Longitudinal Study of Aging* (ELSI-Brazil) conducted between 2015 and 2016 in 70 municipalities across all Brazilian regions. The ELSI-Brazil is a nationally representative population-based cohort study of people aged 50 years or older and is part of an international network of large longitudinal studies on aging called *Health and Retirement Family of Studies* ^18^.

### Ethics

The study was approved by the Brazilian National Research Ethics Committee (CAAE 63725117.9.0000.5091). A written informed consent form was obtained from every participant.

### Sample

The ELSI-Brazil has a sampling process that comprises selection stages combining households, census sectors, and stratification of primary sampling units (municipalities). The sample was divided into four strata, with the first stratum drawn from 4,420 municipalities constituting ≤ 26,700 inhabitants, the second from 951 municipalities constituting 26,701-135,000 inhabitants, the third from 171 municipalities constituting 135,000-750,000 inhabitants, and the fourth from 23 municipalities constituting > 750,000 inhabitants. More details about the sampling process are available in Lima-Costa et al. ^18^. All residents in the selected households aged 50 years and over were invited to participate in the study. The ELSI-Brazil final sample comprised 9,412 individuals ^18^.

### Outcome assessment

The outcome was assessed with the question: “Would you say that your oral health (teeth and gums) is...”, later dichotomized as “good” (very good/good) and “poor” (fair/poor/very poor) ^19^ ^,^ ^20^.

### Intermediate determinants

Covariates were based on self-reports, including age (50 to 59 years, 60 to 69 years, and 70 years and older) ^19^, sex (male/female), schooling (0 to 8 years/9 or more years) ^19^, race (categorized in white and non-white), and wealth.

#### Wealth index assessment

The wealth index was created based on the national population, using a multivariate statistical technique that consists of transforming a set of original variables into another set of variables of equal values, called principal components ^21^. Information on the ownership of durable goods and housing characteristics contained in the household questionnaire was used: access to the Internet, ownership of television, DVD or VCR, cable TV, refrigerator, washing machine, dishwasher, dryer, computer, landline, mobile phone, microwave, air conditioning, motorcycle, car, house with a housemaid, masonry wall, access to running water, street access pavement, presence of bathroom, and family agglomeration (number of rooms in the house divided by the number of residents). The variable was categorized into quintiles, like the other variables, as has been done in previous studies ^22^ ^,^ ^23^.

#### Race assessment

The racial classification was self-reported and based on the classification of the Brazilian Institute for Geography and Statistics (IBGE), which includes whites, mixed-race, blacks, yellow people, and indigenous people ^6^. Due to the low frequency, indigenous (1.06%) and yellow (1.87%) participants were excluded from the current analysis, so that all descriptive and analytical questions turned to the comparison between whites and non-whites. The category of non-whites was formed by black and mixed-race individuals. Based on a strictly statistical point of view, only the socioeconomic similarities between black and mixed-race individuals would justify such aggregation. Although studies have already proposed that the socioeconomic status of mixed-race individuals would be intermediate between blacks and whites, other studies based on more solid empirical evidence have shown little or no difference between the two groups ^24^.

### Proximal determinants

The psychosocial variables included were depression, satisfaction with life, neighborhood trust, and social participation. Depression was assessed by the following question: “Has any physician ever said that you have depression?” (yes/no). To assess satisfaction with life, participants were instructed to think about their level of satisfaction with life and to point to a step ladder with numbers from 1 to 10, with the highest step corresponding to the number 10, representing the maximum satisfaction level, and the lowest step being number 1, representing the lowest satisfaction. This variable was dichotomized as “low satisfaction” (steps from 1 to 5) and “high satisfaction” (step number 6 or higher). To measure trust in the neighborhood the following questions were included: “Do you think you can trust most people in the neighborhood?” (yes/no) and “Do you have friends?” (yes/no), relying on previous studies ^25^. To measure social participation, the following question was included: “In the last 12 months, did you participate in organized social activities (clubs, community or religious groups, community center, senior university, etc.)?” (yes/no) ^19^.

Oral health behavior was assessed by the questions: “Do you use toothbrush?” and “Do you use dental floss?” with yes/no as answer options. The smoking habit was assessed by the question: “Currently, do you smoke (considering smoking industrial cigarettes, straw cigarettes, or other tobacco products such as cigars, cigarillos, pipes, cloves cigarettes, Indian cigarettes, and hookahs)?”. With the answer options: “yes, daily”, for those who smoke every day, at least one of the products; “yes, less than daily”, for those who smoke but not every day; and “no”, for those who do not smoke, not even occasionally. Dental attendance was assessed by the question: “When did you last visit a dentist?” (less than a year ago/in a year or more/never did). Other oral health measures included number of remaining teeth, assessed by: “How many teeth do you have?” with responses categorized considering the functional dentition (presence of 20 teeth or more) as follows: 1 to 9 teeth, 10 to 19 teeth, and 20 or more teeth ^26^. The number of teeth is considered an important oral health indicator ^22^ ^,^ ^26^ ^,^ ^27^.

### Theoretical conceptual model

The theoretical conceptual model sought to follow hypothetical association between social determinants of health and oral health and is strongly supported by a study conducted by Peres et al. ^28^, in 2019.

### Statistical analysis

Since this was a complex sample, all analysis incorporated the sample weight using the *svy* command. Preliminary analyses described the data and presented the prevalence of variables. Crude and adjusted prevalence ratios (PR) were determined, with a 95% confidence interval (95%CI). The associations between self-reported oral health and wealth index were stratified by the individuals’ race. Poisson regression models were used to analyze the direct and indirect effect of the wealth index on self-reported oral health. The results reported were those of Model 2, based on the previous literature that indicates possible hypothetical paths linking race and self-reported oral health ^28^ ^,^ ^29^. All analyses were performed on Stata 14.0 (https://www.stata.com).

## Results

The ELSI-Brazil sample consisted of 9,412 individuals; however, the final sample of this study was composed of 9,365 individuals who completed the self-report measures of oral health. Table 1 shows the population and environmental characteristics of the sample. Most participants were women, aged from 50 to 59 years, non-whites, with less than 8 years of education. Also, most participants did not have a medical diagnosis of depression, reported not having social participation, trust most people in their neighborhood, are satisfied with life, last sought dental services more than a year ago, do not have a smoking habit, use a toothbrush but do not dental floss, and have 20 teeth or more. The total prevalence of white and non-white who reported having poor self-perceived oral health was 41.6% and 48% respectively.

**Table 1:** Sample characteristics and prevalence of poor self-reported oral health by intermediate and proximal determinants. *Brazilian Longitudinal Study of Aging* (ELSI-Brazil).

Characteristics	Weighted %	Poor self-reported oral health Prevalence (95%CI)
Intermediate determinants		
Sex		
Female	54.0	41.8 (39.9-43.7)
Male	46.0	49.7 (47.6-51.7)
Age (years)		
50-59	47.6	51.4 (49.0-53.8)
60-69	29,7	43.8 (41.6-46.0)
≥ 70	22,7	34.9 (32.7-37.2)
Race/Skin color		
White	42.7	41.6 (40.0-43.4)
Non-white	54.3	48.0 (47.1-49.8)
Schooling (years)		
0-8	73.1	46.3 (44.4-48.3)
> 8	26.9	42.9 (40.1-45.7)
Wealth (quintiles)		
1st (poorest)	20.0	47.8 (45.7-49.9)
2nd	20.0	47.4 (45.2-49.6)
3rd	20.0	45.2 (43.0-47.5)
4th	20.0	45.5 (43.2-47.8)
5th (richest)	19.0	39.7 (37.3-42.1)
Proximal determinants		
Depression		
Yes	18.6	51.2 (48.4-54.0)
No	81.4	44.1(42.2-45.9)
Social participation		
Yes	48.7	43.7 (41.5-45.9)
No	51.3	47.0 (44.9-49.2)
Trust status		
Yes	54.6	41.6 (39.8-43.4)
No	45.4	50.2 (47.8-52.6)
Satisfaction with life		
Low	29.0	53.5 (50.9-56.1)
High	70.9	42.1 (40.2-44.0)
Use of dental service		
Less than a year ago	32.6	43.9 (41.8-46.1)
In a year or more	66.0	46.0 (44.0-48.1)
Never used	1.4	51.0 (41.1-60.7)
Smoking habit		
Yes	17.0	44.3 (42.4-46.3)
No	83.0	50.6 (47.8-53.4)
Use of toothbrush		
No	3.2	39.3 (32.2-46.7)
Yes	96.8	45.6 (44.0-47.3)
Use of dental floss		
No	61.6	45.5 (43.7-47.4)
Yes	38.4	45.3 (42.9-47.8)
Number of teeth		
0	29.6	75.3 (73.2-77.3)
1-9	23.3	44.5 (42.1-46.8)
10-19	17.0	38.5 (35.6-41.5)
20 or more	29.9	53.8 (51.1-56.5)

95%CI: 95% confidence interval.

Note: considering the sample weight.

Tables 2 and 3 show the unadjusted and adjusted associations between the wealth index and poor self-reported oral health by Poisson regression models. Model 1 showed the association between wealth index and self-reported oral health adjusted by intermediary determinants. It suggests that, for whites, the wealth index is associated with self-reported oral health, with individuals in the 3rd, 4th, and 5th quintiles having 23%, 20%, and 36% lower prevalence of poor self-reported oral health than those in the poorest quintile. After adjustment by interproximal determinants in Model 2, the association remains. Whites still have better perception of oral health, with individuals in the 3rd, 4th, and 5th quintiles having 25%, 20%, and 39% lower prevalence of poor self-reported oral health than those in the poorest quintile.

**Table 2:** Unadjusted and adjusted association of wealth index variables with poor self-reported oral health in older white Brazilians, determined using Poisson regression. *Brazilian Longitudinal Study of Aging* (ELSI-Brazil).

Wealth index (quintiles)	Unadjusted PR (95%CI)	Model 1 * PR (95%CI)	Model 2 ** PR (95%CI)
1st (poorest)	1.00	1.00	1.00
2nd	0.88 (0.77-1.01)	0.87 (0.75-1.01)	0.90 (0.77-1.07)
3rd	0.82 (0.71-0.94) ***	0.77 (0.66-0.90) ***	0.75 (0.65-0.88) ***
4th	0.84 (0.74-0.96) ***	0.77 (0.66-0.90) ***	0.80 (0.67-0.95) ***
5th (richest)	0.71 (0.62-0.80) ***	0.64 (0.53-0.78) ***	0.61 (0.50-0.75) ***

95%CI: 95% confidence interval; PR: prevalence ratio.

Note: considering the sample weight.

* Adjusted Model 1: for sex, age, and schooling;

** Adjusted Model 2: depression, social participation, trust in the neighborhood, satisfaction with life, use of dental service, smoking, use of floss, use of toothbrush, and number of teeth;

*** Statistically significant differences.

**Table 3:** Unadjusted and adjusted association of wealth index variables with poor self-reported oral health in non-white older Brazilians, determined using Poisson regression. *Brazilian Longitudinal Study of Aging* (ELSI-Brazil).

Wealth index (quintiles)	Unadjusted PR (95%CI)	Model 1 * PR (95%CI)	Model 2 ** PR (95%CI)
1st (poorest)	1.00	1.00	1.00
2nd	1.07 (1.00-1.15) ***	1.05 (0.97-1.13)	1.07 (0.99-1.17)
3rd	0.98 (0.89-1.08)	0.95 (0.86-1.05)	0.99 (0.88-1.11)
4th	0.97 (0.84-1.12)	0.93 (0.81-1.07)	0.94 (0.82-1.08)
5th (richest)	0.84 (0.73-0.96) ***	0.81 (0.70-0.93) ***	0.85 (0.72-0.99) ***

95%CI: 95% confidence interval; PR: prevalence ratio.

Note: considering the sample weight.

* Adjusted Model 1: for sex, age, and schooling;

** Adjusted Model 2: depression, social participation, trust in the neighborhood, satisfaction with life, use of dental service, smoking, use of floss, use of toothbrush, and number of teeth;

*** Statistically significant differences.

For non-white individuals, only those in the 5th quintile showed a 19% lower prevalence of poor self-reported oral health than those in the poorest quintile after adjustment for intermediary determinants in Model 1. After adjustment for interproximal determinants in Model 2, the association remains with the 5th quintile suggesting that these individuals have 15% lower prevalence of poor self-reported oral health than those in the poorest quintile.

## Discussion

This study aimed to identify the association between self-reported oral health and the wealth index among white and non-white adults in Brazil. The findings are in line with the hypothesis of this study, considering that even non-whites belonging to the wealthiest quintiles would have worse self-reported oral health than whites from the same quintile.

The individual socioeconomic level, assessed by wealth, partially explain the racial inequalities in oral health, since these are often related to lower socioeconomic status and the consequent detrimental patterns of health behavior and barriers to dental care ^30^. However, the persistent racial gaps in oral health go beyond that, relying in wider processes such as systemic racism and discrimination ^4^. A study conducted in Brazil ^31^showed that patient skin color influenced the dentist’s choice of treatment, and black patients received generally referrals for cheaper and simpler procedures, which indicates that even the professionals may contribute unconsciously to the replication of racial discrimination. The interaction between socioeconomic barriers and discrimination possibly led to the poorer self-reported oral health among non-whites in this study, and can reaffirm the sociohistorical legacy of the course of action of racial structures ^32^, stereotyping, and stigmatization ^17^.

Privileges linked to the accumulated past of nations for some racial groups can play a crucial role in how individual wealth is distributed and how race can disproportionately influence wealth distribution and consequently health outcomes ^23^. The theory of the race discrimination system assumes a feedback relationships among domains or subsystems. For example, racial residential segregation found in contemporary United States has historically been linked to slavery and is considered a powerful linking force between socioeconomic status and health ^33^. Also, the manifestation of multiple systems of oppression linked to social structures created in the past, such as political marginalization and economic exploitation faced by racial minorities, distorts how people see each other, the attributions one makes about them, and the predictions of their performance, and is the major driving force behind societal imbalances ^3^ ^,^ ^33^. Previous studies often recognize the social, cultural and historical privileges attributed to some specific racial categories and the disadvantage that other people face ^4^ ^,^ ^13^. The uneven distribution of resources and power seems to lead to an unequal interaction between different racial groups ^3^ ^,^ ^4^.

Socioeconomic status, ethnic/racial inequalities, and different oral health outcomes show a strong relationship ^34^. Strategies to minimize this association must be considered by policy makers and managers. Among the strategies are the work with race, to describe the inequitable distribution of adverse dental outcomes, the increase of racial diversity in power spaces, and the construction of an anti-racist narrative ^33^. In addition, the common risk factor approach for planning and implementing techniques to mitigate racial oral health inequities may be a positive method, counting with the interaction between study areas and social sectors ^33^ ^,^ ^35^. Future actions to address inequalities in oral health in middle- and high-income countries require a radical political reorientation to deal with structural and environmental determinants ^29^ ^,^ ^36^. The common risk factor approach is a possible facilitator of a greater integration of oral health in general strategies of health improvement ^29^ ^,^ ^36^.

The findings suggest that non-white individuals in the 5th quintile (the richest) had a better perception of oral health than those in other quintiles. For whites, those in the 5th, 4th, and 3rd quintiles showed a better perception of oral health, differing from the first two quintiles. These findings suggest possible patterns of inequality, such as the marginal exclusion or “bottom inequality”. This pattern is identified when a given intervention reaches most of the population, but fails to reach a less privileged group, such as the quintile with the lowest socioeconomic level ^27^ ^,^ ^37^. This type of inequality is quite prevalent in middle-income countries such as Brazil ^37^. Carrying out studies aimed at measuring and monitoring trends in inequality related to skin color, socioeconomic factors, and oral health are important, fostering policies with more efficient approaches for improving population health and reducing such inequalities ^38^.

These findings should be interpreted with caution due to some limitations. The cross-sectional design limits the scope of causal inferences, highlighting the need for prospective studies. Also, perception bias is a possibility, considering the evidence that people are unconsciously influenced, in the decision-making process, by past experiences. Considering that race is a socially constructed concept and is mediate by discrimination is important. Therefore, the concept of race is not fixed or inherent, while many individuals identify themselves with a single racial category, others can identify themselves as biracial or multiracial and self-identification can evolve over time assuming new social or political meanings ^2^. Regarding use of self-reported oral health, although methodologically challenging, there are acceptable sensitivity and specificity values for self-reported oral health and oral health conditions ^20^. This suggests that the questions can be used for this purpose. The strength of this study is the assessment of a large population-based data, representative of all Brazilian regions, including cities of different sizes.

In conclusion, it is suggested that the wealth index has a greater effect on white individuals when associated with the self-reported oral health. These findings reinforce the patterns of health inequality, since the most privileged individuals showed better oral health outcomes than their less privileged peers. This study highlights the importance of developing policies to combat racial inequalities for the Brazilian older population.
